# Sibling cases of gross hematuria and newly diagnosed IgA nephropathy following SARS-CoV-2 vaccination

**DOI:** 10.1186/s12882-022-02843-2

**Published:** 2022-06-21

**Authors:** Yuri Uchiyama, Hirotaka Fukasawa, Yuri Ishino, Daisuke Nakagami, Mai Kaneko, Hideo Yasuda, Ryuichi Furuya

**Affiliations:** 1 Renal Division, Department of Internal Medicine, Iwata City Hospital, 512-3 Ohkubo, Iwata, Shizuoka 438-8550 Japan; 2 First Department of Medicine, Hamamatsu University School of Medicine, Hamamatsu, Shizuoka Japan

**Keywords:** COVID-19, Gross hematuria, IgA nephropathy, SARS-CoV-2 vaccination, Sibling

## Abstract

**Background:**

Severe acute respiratory syndrome coronavirus 2 (SARS-CoV-2) vaccination has become a major part of the strategy to reduce Coronavirus disease 2019 (COVID-19) numbers worldwide. To date, vaccinations based on several mechanisms have been used clinically, although relapse of existent glomerulonephritis presenting as gross hematuria, and occurrence of *de novo* glomerulonephritis have been reported.

**Case presentation:**

We report the first sibling cases newly diagnosed as immunoglobulin A (IgA) nephropathy after the second dose of SARS-CoV-2 vaccination. 15- and 18-year-old men presented with gross hematuria following the second dose of SARS-CoV-2 vaccine (Pfizer, BNT162b2) received on the same day. Pathological findings of each kidney biopsy specimen were consistent with IgA nephropathy. Gross hematuria in both cases spontaneously recovered within several days.

**Conclusions:**

These cases indicate that SARS-CoV-2 vaccination might trigger *de novo* IgA nephropathy or stimulate its relapse, and also highlight the necessity of understanding the immunological responses to the novel mRNA vaccines in patients with kidney diseases.

## Background

Coronavirus disease 2019 (COVID-19) pandemic is a public health emergency all over the world and its effective control is expected by the execution of a global vaccination strategy [1]. Recently, several types of vaccines against severe acute respiratory syndrome coronavirus 2 (SARS-CoV-2) developed and progressively used worldwide [2–5].

On the other hand, several studies have reported the appearance of gross hematuria following messenger ribonucleic acid (mRNA)-based SARS-CoV-2 vaccination in patients with glomerulonephritis or in patients developing *de novo* glomerulonephritis post vaccination, especially those with immunoglobulin A (IgA) nephropathy [6–14].

In this manuscript, we report the first sibling cases of newly diagnosed IgA nephropathy who developed gross hematuria following the second dose of SARS-CoV-2 vaccination.

## Case presentation

### Patient 1

A 15-year-old Japanese man with a 6-months history of microscopic hematuria was referred to our hospital due to gross hematuria 1 day after receiving the second dose of SARS-CoV-2 vaccine (Pfizer, BNT162b2). He also had fever and myalgia. He had no prior history of COVID-19 infection, nor gross hematuria after any other infections. He had no family medical history of kidney diseases.

Physical examination on admission revealed body temperature of 37.7 °C, pulse rate of 72 beats/min, blood pressure of 115/64 mmHg and no costovertebral angle tenderness.

Laboratory findings revealed serum creatinine of 0.97 mg/dL, estimated glomerular filtration rate (eGFR) of 92 mL/min/1.73 m^2^, and urinalysis revealed numerous red blood cells and moderate proteinuria Table 1. There was no morphological abnormality of his kidneys in the imaging of computed tomography scan.

**Table 1:** Clinical characteristics and laboratory findings on initial presentation

Variables	Patient 1	Patient 2
Age, years	15	18
Gender	Male	Male
Duration to hematuria after vaccination	1 day	2 days
Body mass index, kg/m^2^	22.0	18.2
Hemoglobin, g/dL	13.8	15.7
Albumin, g/dL	5.3	5.2
LDL cholesterol, mg/dL	51	111
Blood urea nitrogen, mg/dL	10	11
Serum creatinine, mg/dL	0.97	0.82
eGFR, mL/min/1.73m^2^	92	105
Serum sodium, mEq/L	139	139
Serum potassium, mEq/L	4.0	4.2
CRP, mg/dL	0.55	0.52
Serum HCO3^-^, mEq/L	28.4	25.9
Urinary protein, (-) - (3+)	3+	1+
Urinary protein, g/gCreatinine	0.9	0.4
Urinary occult blood, (-) - (3+)	3+	3+
Urinary ß2-microglobulin, μg/L	125	72
Urinary NAG, IU/L	1.8	3.0

*Abbreviations*: *CRP* C-reactive protein, *eGFR* estimated glomerular filtration rate, *HCO*_*3*_^*-*^ bicarbonate, *LDL* low-density lipoprotein, *NAG* N-acetyl-glucosaminidase

**Fig. 1:**
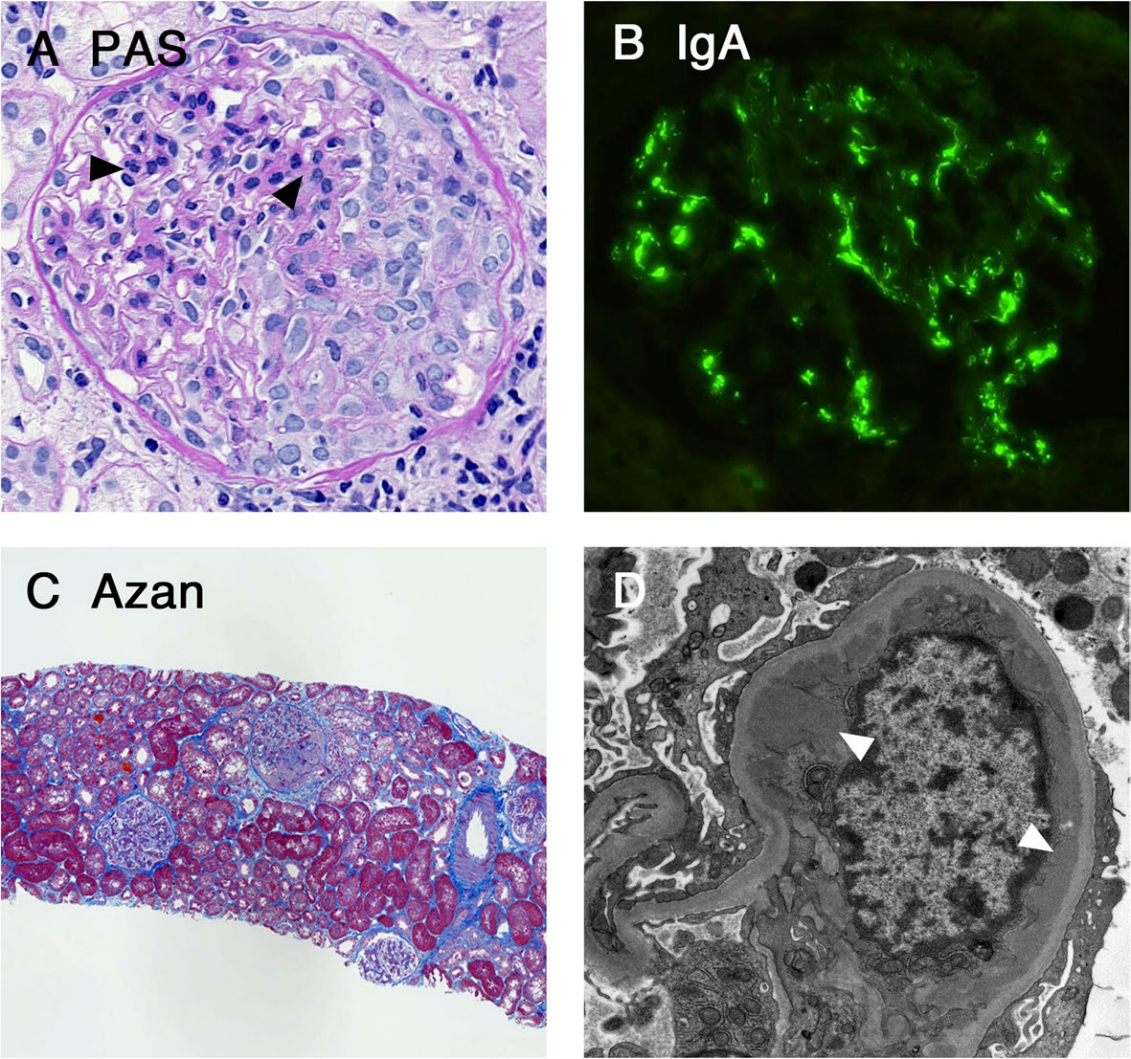
Findings of the kidney biopsy specimens from patient 1. (**A**) Glomerular mesangial expansion and hypercellularity was observed (arrowheads). A cellular crescent formation was also observed. (PAS staining, × 400). (**B**) Glomerular mesangial deposits for IgA antisera were observed (immunofluorescence study, × 400). (**C**) Approximately 10-20% tubulointerstitial fibrosis of the cortex was observed (Azan staining, ×100). (**D**) Ultrastructural evaluation revealed the electron-dense deposits in the mesangium (arrowheads, transmission electron microscopy, x4000). Abbreviation: IgA, Immunoglobulin A; PAS, Periodic acid Shiff

The kidney biopsy was performed 7 days after the onset of gross hematuria. Among 21 glomeruli sampled, there were no globally sclerotic glomeruli. All glomeruli showed diffuse and mild mesangial expansion and hypercellularity, and 1 of which had a cellular crescent formation. There was approximately 10-20% tubulointerstitial fibrosis of the cortex and no arterio- and arteriolosclerosis. Immunofluorescence revealed global mesangial staining of IgG (1+), IgA (2+), IgM (±), C3 (1+), C4 (-) C1q (-) and fibrinogen (±). Ultrastructural examination revealed electron dense deposits in the mesangium and mild podocyte foot process effacement (Fig. 1). Pathological features were consistent with IgA nephropathy and the Oxford MEST-C classification was M1E0S0T0C1 [15]. His gross hematuria spontaneously resolved within 6 days without any treatment, although his microscopic hematuria and proteinuria persisted after that.

### Patient 2

An 18-year-old Japanese man with a 3-years history of microscopic hematuria was referred to our hospital due to gross hematuria 2 days after receiving the second dose of the SARS-CoV-2 vaccine (Pfizer, BNT162b2). In fact, he was the brother of patient 1 and had received the vaccination on the same day. He also had fever and general malaise. He had no prior history of COVID-19 infection, nor gross hematuria after any other infections. He had no family medical history of kidney diseases.

Physical examination on admission revealed body temperature of 38.6 °C, pulse rate of 83 beats/min, blood pressure of 131/76 mmHg and no costovertebral angle tenderness.

Laboratory findings revealed serum creatinine of 0.82 mg/dL, eGFR of 99 mL/min/1.73 m^2^, and urinalysis revealed numerous red blood cells and mild proteinuria Table 1. There was no morphological abnormality of his kidneys in the imaging of computed tomography scan.

**Fig. 2:**
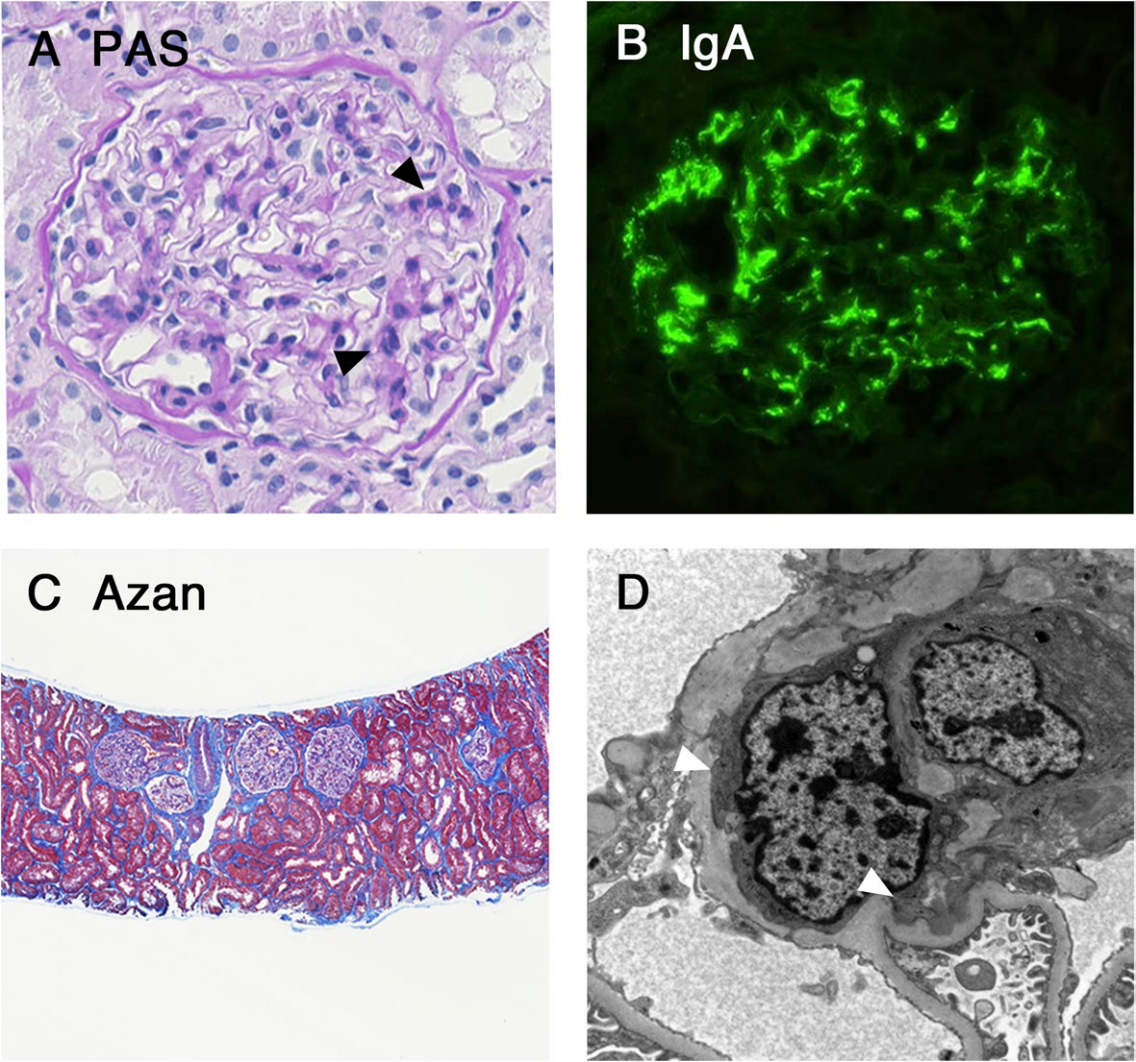
Findings of the kidney biopsy specimens from patient 2. (**A**) Glomerular mesangial expansion and hypercellularity was observed (arrowheads). (PAS staining, × 400). (**B**) Glomerular mesangial deposits for IgA antisera were observed (immunofluorescence study, × 400). (**C**) Approximately 10% tubulointerstitial fibrosis of the cortex was observed (Azan staining, ×100). (**D**) Ultrastructural evaluation revealed the electron-dense deposits in the mesangium (arrowheads, transmission electron microscopy, ×4000). Abbreviation: IgA, Immunoglobulin A; PAS, Periodic acid Shiff.

The kidney biopsy was performed 3 months after the onset of gross hematuria. Among 67 glomeruli sampled, there were no globally sclerotic glomeruli. All glomeruli showed diffuse and mild mesangial hypercellularity without crescent formation. There was approximately 10 % tubulointerstitial fibrosis of the cortex and no arterio- and arteriolosclerosis. Immunofluorescence revealed global mesangial staining of IgG (±), IgA (2+), IgM (±), C3 (1+), C4 (-) C1q (±) and Fibrinogen (±). Ultrastructural examination revealed electron dense deposits in the mesangium and mild podocyte foot process effacement (Fig. 2). Pathological features were consistent with IgA nephropathy and the Oxford MEST-C score was M1E0S0T0C0 [15]. His gross hematuria spontaneously resolved within 7 days without any treatment, and his microscopic hematuria and proteinuria also disappeared gradually.

## Discussion and conclusions

To our best knowledge, this is the first report showing sibling cases that developed gross hematuria and were newly diagnosed as IgA nephropathy following the SARS-CoV-2 vaccination.

Recent reports indicate an association between gross hematuria and the SARS-CoV-2 vaccination in patients with glomerulonephritis, especially those with IgA nephropathy [6–14]. To date, vaccinations based on several mechanisms have been used clinically, although gross hematuria was only reported after receiving the mRNA vaccines [2–5]. The BNT162B2 (Pfizer) and the mRNA-1273 (Moderna) vaccines employ a purified mRNA lipid nanoparticle-encapsulated platform. This novel mRNA-based platform induces stronger antigen-specific cluster of differentiation (CD) 4+ and CD 8+ T cell responses [16]. Because the CD 4+ and CD 8+ T cells activated by vaccination produce several proinflammatory cytokines, including interferon-γ and tumor necrosis factor-α, these vaccines might exacerbate immune-mediated glomerular diseases or cause *de novo* glomerulonephritis including IgA nephropathy [6].

One notable point in the present cases is that microscopic hematuria has already existed before the vaccination. In addition, the chronic histopathologic features such as tubulointerstitial damage indicate the possibility that the immune responses to vaccination exacerbated a pre-existing undiagnosed IgA nephropathy in our cases.

Another notable point is the association between the pathogenesis of IgA nephropathy and Toll-like receptors (TLRs), which are a family of immune receptors whose activation is important for the mucosal immune responses [17]. Increased amounts of abnormally glycosylated IgA1 have been thought to be the first hit in the development of IgA nephropathy [18]. Recently, it was reported that TLR7 recognizes endogenous or exogenous single-strand RNAs and is involved in the production of abnormally glycosylated IgA1 [19]. Taken together, it is possible that the mRNA vaccination causes the production of abnormally glycosylated IgA1 via TLR signaling and is related to the exacerbation of IgA nephropathy, at least partially [20].

In conclusion, we report the first sibling cases of newly diagnosed IgA nephropathy who developed gross hematuria following the second dose of SARS-CoV-2 vaccination. These cases indicate that SARS-CoV-2 vaccination might stimulate the relapse or trigger *de novo* IgA nephropathy. On the other hand, it remains to be unknown that gross hematuria developed in our patients was caused by chance or due to the genetic related etiology. Further studies are needed to identify how this postvaccination setting can develop and how we should manage these patients with IgA nephropathy.

## Data Availability

The datasets used and/or analyzed are available from the corresponding author upon reasonable request.

## Abbreviations

CD: cluster of differentiation
COVID-19: coronavirus disease 2019
CRP: C-reactive protein
eGFR: estimated glomerular filtration rate
HCO_3_^-^: bicarbonate
IgA: immunoglobulin A
LDL: low-density lipoprotein
mRNA: messenger ribonucleic acid
NAG: N-acetyl-glucosaminidase
PAS: Periodic acid Shiff
SARS-CoV-2: severe acute respiratory syndrome coronavirus 2
TLR: toll-like receptor
